# Design of Airborne Large Aperture Infrared Optical System Based on Monocentric Lens

**DOI:** 10.3390/s22249907

**Published:** 2022-12-16

**Authors:** Jiyan Zhang, Teng Qin, Zhexin Xie, Liting Sun, Zhengyu Lin, Tianhao Cao, Chentao Zhang

**Affiliations:** 1 Fujian Key Laboratory of Optoelectronic Technology and Devices, Xiamen University of Technology, Xiamen 361024, China; 2 Department of Instrumental and Electrical Engineering, Xiamen University, Xiamen 361000, China

**Keywords:** optical design, mid-wave infrared, monocentric lens, large aperture, sensor array

## Abstract

Conventional reconnaissance camera systems have been flown on manned aircraft, where the weight, size, and power requirements are not stringent. However, today, these parameters are important for unmanned aerial vehicles (UAVs). This article provides a solution to the design of airborne large aperture infrared optical systems, based on a monocentric lens that can meet the strict criteria of aerial reconnaissance UAVs for a wide field of view (FOV) and lightness of airborne electro-optical pod cameras. A monocentric lens has a curved image plane, consisting of an array of microsensors, which can provide an image with 368 megapixels over a 100° FOV. We obtained the initial structure of a five-glass (5GS) asymmetric monocentric lens with an air gap, using ray-tracing and global optimization algorithms. According to the design results, the ground sampling distance (GSD) of the system is 0.33 m at 3000 m altitude. The full-field modulation transfer function (MTF) value of the system is more than 0.4 at a Nyquist frequency of 70 lp/mm. We present a primary thermal control method, and the image quality was steady throughout the operating temperature range. This compactness and simple structure fulfill the needs of uncrewed airborne lenses. This work may facilitate the practical application of monocentric lens in UAVs.

## 1. Introduction

The development of drone technology has accelerated over the last few decades. It has thus far been used in industries such as the military [1,2,3], agriculture [4,5,6], and the news media [7,8]. Likewise, uncrewed airborne camera systems have advanced quickly. Unmanned reconnaissance aircraft play a vital role in modern military operations, due to their small size, high flexibility, and long-lasting endurance. New camera systems must be compact, lightweight, power-efficient, and meet resolution and wide-field requirements. An infrared reconnaissance system is a passive, non-contact detection and recognition system with good concealment and immunity from electromagnetic interference. It can accurately track thermal targets from a long distance, accurately guide, and truly achieve 24-h all-weather monitoring. Infrared reconnaissance systems have been applied in the following fields: military reconnaissance, strategic strike, target tracking, and airspace boundary patrol [9,10,11]. Airborne optoelectronic pods serve as the “eyes” of unmanned reconnaissance aircraft. The infrared optical system is one of the essential components of airborne pod systems, for performing all-weather target identification and detection under challenging weather conditions, such as in dense fog or at night [12,13].

There are no strict limitations to the size, weight, and power of onboard cameras in conventional crewed aircraft. Nevertheless, these factors have become crucial. New camera systems for UAVs must be smaller, more compact, lighter in weight, and consume less power, while maintaining a high resolution and a wide FOV for wide area persistent surveillance. A traditional fisheye lens would fulfill the requirement of a wide FOV, but its structure is very bulky relative to its aperture size and the field curvature also needs to be corrected. With conventional lenses, it is still very difficult to achieve diffraction limited performance over a wide-field, while maintaining a low F number. To be integrated into a UAV, a camera must meet the resolution and FOV standards, while being lighter, smaller, and requiring less power [14,15,16,17].

This paper presents a compact infrared optical system based on a monocentric structure, to meet the requirements of UAVs for the small size and wide FOV of the optical systems. This paper is organized as follows. In Section 2, a simplified model of the monocentric lens is discussed. The design principle and application of monocentric lenses are also discussed. In addition, the initial structure of the system is obtained using ray tracing and global optimization algorithms. The relationship between the parameters of the monocentric lens and the imaging sensor is considered. In Section 3, the system optimization and results analysis based on the parameters presented in Section 2 are provided. The system optimization results showed that the system has good image performance and can fulfill the requirements of a UAV camera. In Section 4, heatless processing and tolerance analyses are presented. This description is accompanied by a detailed discussion of the primary thermally controlled method. A tolerance analysis demonstrates the lens’ machinability, which can meet the requirements for industrial production. Section 5 presents our conclusions.

## 2. Optical System Design

### 2.1. Monocentric Lenses

**Figure 1:**
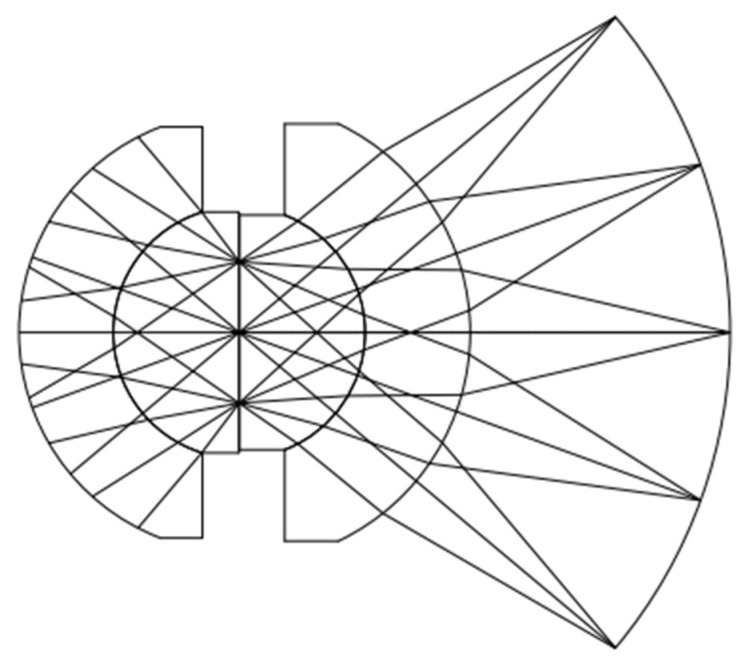
Diagram of the monocentric lens with ray traces.

Figure 1 is a diagram of the monocentric lens with ray traces. The lens is characterized by all element surfaces having a common curvature center. An aperture stop is placed at the center of the curvature. Unlike an ordinary lens, monocentric systems have no tightly defined axis. There are no off-axis aberrations, e.g., coma, astigmatism, and lateral chromatic aberration, compared to previous systems. Only spherical aberration, axial chromatic aberration, and spherochromatic are present. The difficulty of design is significantly lowered [18,19,20,21,22,23,24]. A monocentric lens provides a compact system structure, while obtaining a large FOV and greatly reduces the total length of the system.

**Figure 2:**
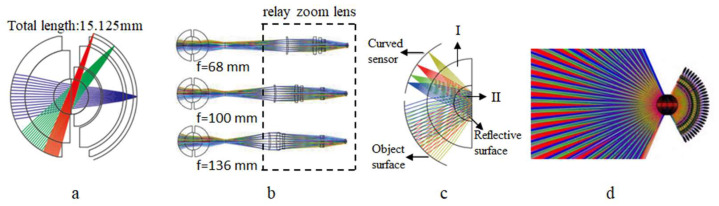
Monocentric lenses for different applications (**a**) miniaturized ultra-wide-angle surveillance lens, (**b**) wide-field infrared zoom lens, (**c**) reflective mobile phone monocentric lens, (**d**) ultra-low-altitude remote sensing monocentric lens.

Scholars have studied many applications of monocentric lens design; for example, monocentric mobile phone lenses, monocentric surveillance cameras, monocentric infrared lenses, monocentric aerial photography lenses, etc. In 2018, Xiangyue Meng et al. [25] designed a miniaturized ultra-wide-angle surveillance lens based on a monocentric lens (Figure 2a). In 2019, Wei Yang et al. [26] designed a wide FOV infrared zoom imaging system based on a monocentric lens (Figure 2b). In 2021, Yang Wang et al. [27] designed a monocentric reflective mobile phone lens using the principle of a monocentric lens (Figure 2c). In 2021, Jiahui Li et al. [28] designed an ultra-low-altitude remote sensing camera based on a monocentric lens (Figure 2d). The monocentric lens achieved a wide FOV, while maintaining a compact system structure.

### 2.2. Lens Structure Selection and Design

**Figure 3:**
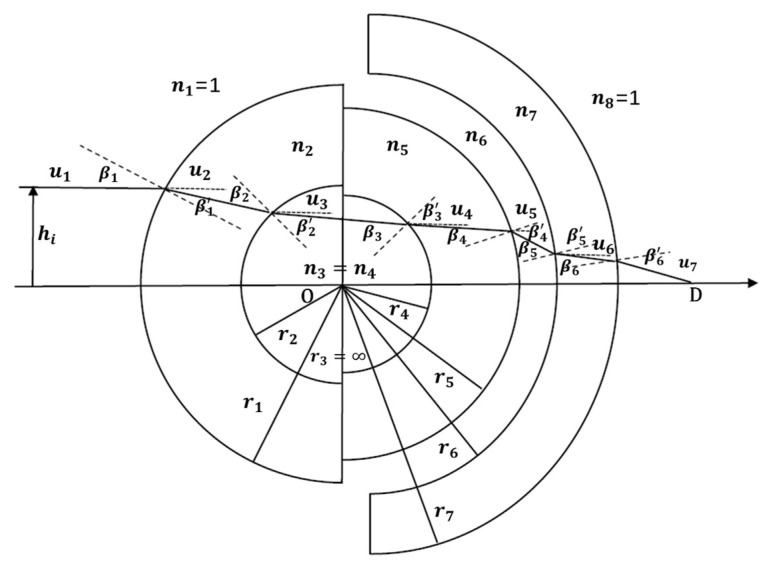
Optical path diagram of the monocentric lens.

This paper substituted a five-glass asymmetric structure with an air gap for the conventional one-glass symmetric (1GS) and two-glass symmetric (2GS) monocentric symmetric structures, to better meet the requirements of the use environment and imaging quality of the optical system. Although 5GS is more complicated than a monocentric symmetric structure, it has more degrees of freedom and is more flexible in terms of design; moreover, the spherical aberrations and longitudinal chromatic aberrations are more effectively corrected [29]. With point O representing the spherical center of the lenses and point D representing the image point, Figure 3 shows the monocentric lens optical path tracing in the 5GS structure. As shown in the Figure, this five-glass air-gap asymmetric geometry improves the performance of the extended spectral bandwidth, with a larger aperture and longer focal length. An air gap was introduced between the fourth and fifth glasses, as this is a common method for controlling spherical and chromatic aberrations [30].

After choosing the type of lens structure, the following stage was to choose the material and calculate the radius of curvature of each lens surface per the design specifications. The optical design parameters are displayed in Table 1, and the global optimization algorithm was used to solve the initial structure of the lens using the design specifications:

(1) $$r_{i}=\frac{h_{i}\left(n_{i+1}-n_{i}\right)}{n_{i+1}u_{i+1}-n_{i}u_{i}}$$

As shown in Figure 3, $h_{i}$ is the height of the incident ray, $n_{i}$ is the refractive index at the *i*th surface, $u_{i}$ is the angle between the ray and the optical axis, and $r_{i}$ is the radius of curvature of each surface of the lens. Ray tracing was performed surface by surface. At each step, the angle u between the ray and the next surface, as well as the ray height h of the next surface, can be obtained from Equations (1)–(3):

(2) $$u_{i+1}=\frac{h_{i}\left(n_{i+1}-n_{i}\right)}{n_{i+1}r_{i}}+\frac{n_{i}u_{i}}{n_{i+1}}$$

(3) $$h_{i+1}=h_{i}-u_{i+1}b_{i}$$

where $b_{i}=\left|r_{i}-r_{i+1}\right|$ is the thickness of the glass, which is equal to the difference between the radius of curvature of the two surfaces. Given the edge ray $u_{1}=0$, the relationship between the focal length and the radius of curvature is given as [31]:

(4) $$f_{i}=\frac{r_{i}r_{i+1}n_{i+1}n_{i+2}}{n_{i+1}^{2}\left(r_{i+1}-r_{i}\right)+n_{i+1}\left(n_{i+2}r_{i}-n_{i}r_{i+1}\right)-d_{i}}$$

(5) $$f=\frac{f_{i}f_{i+1}}{f_{i}+f_{i+1}-d_{i}}$$

The merit function is set as the evaluation function of the optimization algorithm system, to correct the spherical, axial chromatic aberrations, and spherochromatism. Its algebraic expression is $W=\left|W_{040}\right|+\left|W_{020}\right|$, where $W_{040}$ and $W_{020}$ represent the spherical aberration and defocus coefficient, respectively, which are shown in Equations (6) and (7) [32], $n_{im}$ is the image spatial refractive index in Equation (8), $R_{t}$ is the tangential image surface radius, and $R_{s}$ is the sagittal image surface radius:

(6) $$W_{040}=\frac{1}{8}\sum_{k=1}^{7}h_{k}{\left[\frac{\left(u_{k+1}-u_{k}\right)n_{k+1}n_{k}}{n_{k}-n_{k+1}}\right]}^{2}\left(\frac{u_{k+1}}{n_{k+1}}-\frac{u_{k}}{n_{k}}\right)$$

(7) $$W_{020}=\frac{1}{2}\sum_{K=1}^{7}\frac{\left(\frac{1}{n_{k}}-\frac{1}{n_{k}}\right)}{r_{k}}+C$$

(8) $$C=\frac{1}{4n_{im}}\left(\frac{1}{R_{t}}-\frac{1}{R_{s}}\right)$$

To obtain more accurate initial parameters of the lens, for an arbitrary incidence height of rays, we have:

(9) $$\beta_{1}=\sin^{-1}\left(\frac{h}{r_{1}}\right)$$

(10) $$\beta_{1}^{\prime }=\sin^{-1}\left(\frac{h}{r_{1}n_{2}}\right)$$

where $\beta_{1}$ and $\beta_{1}^{\prime }$ are the incident angle and refraction angle, respectively. The angle of incidence and refraction of each surface can be obtained according to Snell’s law and the trigonometric function theorem to obtain the expression for $\bar{OD}$ shown in Equation (11). The longitudinal aberration $\Delta X(h_{i})$ is shown in Equation (12), setting the optimization criterion Q [33], where $p_{j}$ is the reduced array of light heights at the pupil and $\lambda_{j}$ is the wavelength used for weighting:

(11) $$\begin{array}{r} \bar{OD}=h/\sin\left\{\sin^{-1}\left(\frac{h}{r_{1}}\right)+\sin^{-1}\left(\frac{h}{r_{2}n_{2}}\right)+\sin^{-1}\left(\frac{h}{r_{4}n_{5}}\right)+\sin^{-1}\left(\frac{h}{r_{4}}\right)\right.\\ +\;\sin^{-1}\left(\frac{h}{r_{6}n_{7}}\right)+\sin^{-1}\left(\frac{h}{r_{7}}\right)-\sin^{-1}\left(\frac{h}{r_{1}n_{2}}\right)-\sin^{-1}\left(\frac{h}{r_{2}n_{3}}\right)\\ \left.-\;\sin^{-1}\left(\frac{h}{r_{4}n_{4}}\right)-\sin^{-1}\left(\frac{h}{r_{5}n_{5}}\right)-\sin^{-1}\left(\frac{h}{r_{6}}\right)-\sin^{-1}\left(\frac{h}{r_{7}n_{7}}\right)\right\} \end{array}$$

(12) $$\Delta X(h_{i})=\bar{OD}\left(h_{i}\right)-f$$

(13) $$\begin{array}{ll} Q & ={\sum_{i=1}^{6}\sum_{j=1}^{3}\left[p_{j}\frac{\Delta X\left(h_{i}\right)}{\lambda_{j}}\right]}^{2}+{\left[\Delta X\left(h_{1},\lambda_{1}\right)-\Delta X\left(h_{1},\lambda_{3}\right)\right]}^{2}\\ & +\;{\left[\Delta X\left(h_{3},\lambda_{1}\right)-\Delta X\left(h_{3},\lambda_{3}\right)\right]}^{2}+{\left[\Delta X\left(h_{6},\lambda_{1}\right)-\Delta X\left(h_{6},\lambda_{3}\right)\right]}^{2}\cdot \end{array}$$

The mid-infrared band has fewer glass types than the visible to near-infrared band, and the number of glass combinations produced by the system is much smaller than the prior group of glass combinations, significantly reducing the time to solve problems. The lens radius of curvature r, refractive index n, and incident light height h were obtained with the above algorithm using a computer to obtain the best glass combination that satisfied the design parameters.

### 2.3. Sensor Selection

**Figure 4:**
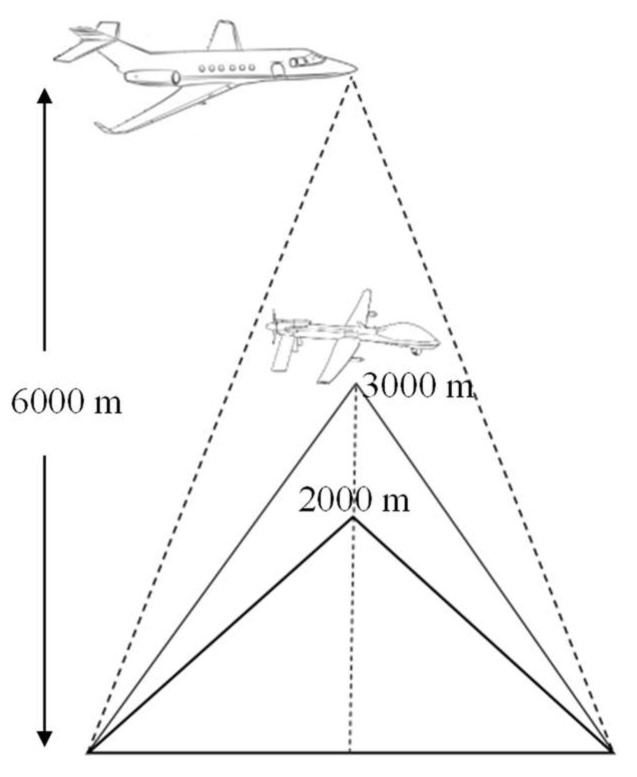
Relationship between working altitude and FOV of a UAV.

On the one hand, monocentric lenses entail the problem of demanding sensor requirements while obtaining a large image plane. At the current state of sensor development, there is no optical sensor that can meet the scale and curvature requirements of this design system. On the other hand, as shown in Figure 4, crewed aircraft generally fly at an altitude of at least 6000 m above the ground, while small reconnaissance UAVs generally work between 2000 m and 3000 m above the ground. This lower altitude flight requires that the optical system is able to provide a wider FOV to cover the same monitoring area. In contrast, wide-area persistent surveillance requires the reconnaissance camera system to provide continuous high-resolution images over a large region.

(14) $$\sigma =\frac{0.61\lambda }{NA}$$

(15) $$\frac{GSD}{\sigma }=\frac{H}{f}$$

Formulas (14) and (15) were calculated to obtain the ground sampling distance (GSD), where H is the height to the ground, NA is the numerical aperture, which is 0.452, λ=5 μm. At a flight altitude of 3000 m, the GSD of the monocentric lens is 0.33 m ($f^{\prime }=63.5\;mm$). The designed system needs to provide a large area of persistent coverage (3 km diameter or more) and at a high resolution (0.33 m GSD) throughout the region of interest. This curved focal plane makes sensor providers increase the pixel count of their sensor’s detector array. The number of effective pixels needed is given by the Formula (16), and each frame of an image requires approximately 368 million pixels, while *P* is the number of effective pixels:

(16) $$P=\pi {\left(H\times \tan50^{{}^{\circ}}\right)}^{2}\left(\frac{1}{GSD^{2}}\right)=\pi {\left(3000\times \tan50^{{}^{\circ}}\right)}^{2}\left(\frac{1}{{\left(0.33\right)}^{2}}\right)=3.68\times 10^{8}$$

**Figure 5:**
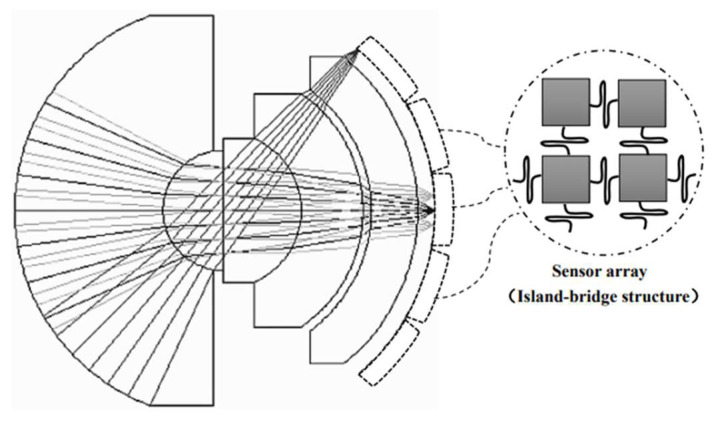
Sensor array diagram.

According to traditional design techniques, this vast pixel array, especially for the infrared spectrum, requires a large detector format and an associated large sensor form factor. Achieving such a sizeable infrared sensor format with a single sensor is difficult. It is well known that a monocentric lens produces a curved focal plane; therefore, the performance of an image system will be greatly enhanced by utilizing an image detector that matches the curved focal surface, and configuring a matched system is not yet easy or cost effective. Using a mosaic of smaller individual detector arrays is more feasible. Combined with the island bridge structure studied in the literature [34], an array was formed using a smaller detector with individual sensors, and the SBF246 mid-infrared sensor from Lockheed Martin with 2048 × 2048 image elements and 7 μm image element spacing was selected. According to the size of the image plane and the size of the sensor, a 10 × 10 array composed of 100 SBF246 can be used to achieve full coverage of the image plane, while this array meets the pixel requirements of Equation (16). This method enables a hemispherical focal plane array, as shown in Figure 5, with curvature fine-tuning enabled by stretching of the bridge axis and elastic substrate. This approach also provides superior situational awareness, allowing small sensors to simultaneously interrogate the area of interest, thereby manipulating the UAV to achieve a more accurate wide-area persistent surveillance pattern.

## 3. System Optimization and Results Analysis

### 3.1. System Optimization Design

The system was optimized using the optical design software of Zemax (Fujian Fotec Optoelectronics Co., Ltd., Fuzhou, China), based on the initial structure, to further improve the imaging quality. The initial structure data of the lens were input to design the monocentric structure; limiting conditions such as the lens interval, focal length, back intercept, and system total length were set; the infinite radius of the curvature of the two asymmetric central lenses and the small- and medium-sized lenses was set as the aperture stop; optimization variables were chosen; and design optimization was conducted. Design optimization was carried out after selecting the optimization factors. We continued by demonstrating the systematic optimization of the lenses using the following process:

(1) Modify the initial structure and make the structural parameters variables.
(2) Set restrictions on the lenses’ thickness, total system length, focal length, back focal length, F number, and wavelength. The thickness of the lens is set to be more than 10 mm and less than 50 mm, due to the restrictions of the airborne large-aperture infrared system size and optical processing. The back focal length is set to be greater than 5 mm.
(3) Use the operands to control the incidence angles to reduce vignetting. The total system length of the lens also needs to be controlled with corresponding operands.
(4) Set the aperture stop at the center of the lens. The matching mold should be made in accordance with the location of the aperture stop during actual manufacture and processing.
(5) A glued surface is added to facilitate processing, assembly, and adjustment. The two hemispherical surfaces should then be joined using the adhesive NOA 61, which can avoid absorption on the surfaces. NOA 61 can withstand temperatures from −15 °C to 60 °C before aging when used for glass bonding. After aging, it can withstand temperatures from −150 °C to 125 °C. The transmissivity of the adhesive reaches 85% in the mid-wave infrared.
(6) Compute and minimize the wavefront deformation to find an approximate surface radii for a valid glass combination to correct for axis chromatism and spherical aberrations.

**Figure 6:**
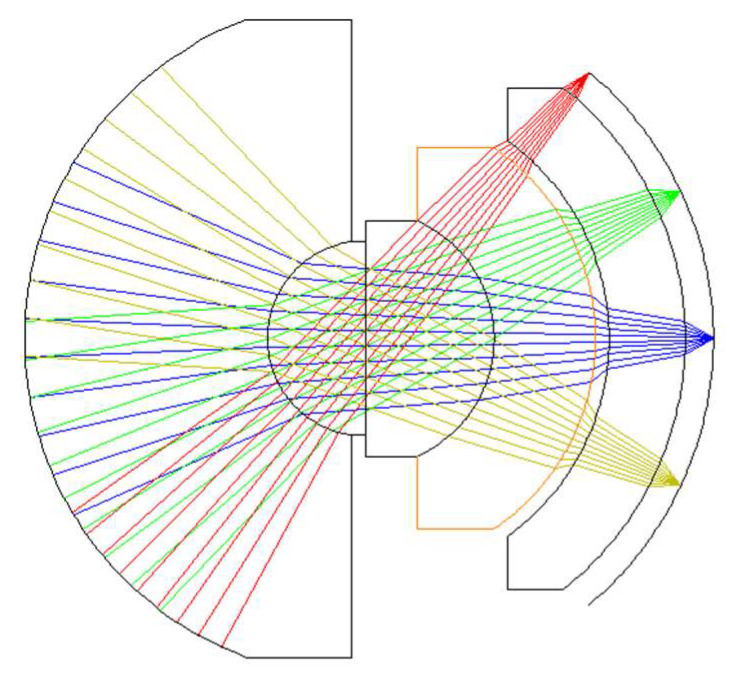
Structure of the designed system.

The optimization results are shown in Figure 6. The system comprises five lenses with the same center curvature and asymmetry. The first lens is GERMANIUM, which has a relatively high refractive index and optical dispersion. The second, third, and fourth lenses are sulfur glass, AMTIR1, and sulfur glass zinc sulfide (ZNS_BROAD), respectively, each with a relatively low refractive index and optical dispersion. The fifth lens is IG2 sulfur glass, which has a high refractive index. Table 2 shows the detailed structural parameters of the optimally designed system. The system has a total length of 124 mm, an F-number of 1, an effective focal length of 63.5 mm, and a full FOV of 100°. The optical system has a length that is comparable to a standard SLR lens. This lens has the benefits of a compact structure and miniaturization when compared to conventional infrared aerial monitoring lenses. The system has a vast monitoring range, due to its wide-field. At an elevation of 3000 m, the diameter of the monitoring area is 7150 m. In such a large monitoring region, it is estimated that the GSD is 0.33 m.

### 3.2. System Optimization Results

**Figure 7:**
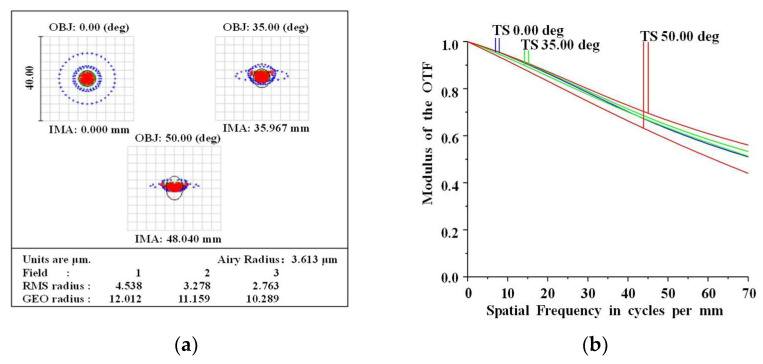
Imaging performance of the monocentric lens for 3000 m. (**a**) MTF curve, (**b**) Spot diagram.

Modulation transfer function (MTF) and spot diagrams were used for aberration performance analysis of the optimized system. Airy spots are large and oval in shape, due to various off-axis aberrations and vignetting of the monocentric lens. A spot diagram is shown in Figure 7a. The relative aperture of the off-axis FOV falls in cosine as the FOV increases. The aperture stop, which causes the elliptical shape of the Airy spot in the off-axis FOVs, is the limiting factor of the system. As can be observed, the imaging quality reaches close to diffraction limited performance, and the imaging performance is uniform across all FOVs. The optical system has good energy collection in various FOVs, as shown in Figure 7a. The Airy spot radius is 3.613 μm, and the RMS spot radius of the system is 4.538 μm, which is within the design error acceptability range and meets the design requirements.

The pixel pitch of the curved sensor is 7 μm. The formula N = 1000/(2a) was used to obtain the Nyquist frequency (N is Nyquist frequency, a is the pixel size). Figure 7b shows the MTF curves of the optical system. Due to the presence of vignetting in the off-axis FOV, there is a shift in the MTF curve as the FOV increases. It can be seen that at 1/2 Nyquist frequency of 35 lp/mm, the MTF value in the 0.7 FOV is greater than 0.78, and the MTF value in the full FOV is greater than 0.7. At the Nyquist frequency of 70 lp/mm, the MTF value in the 0.7 FOV is greater than 0.55, and MTF value in the full FOV is greater than 0.45. The MTF of Figure 7b is favorable, and the lens can achieve fairly good performance up to 70 lp/mm.

**Figure 8:**
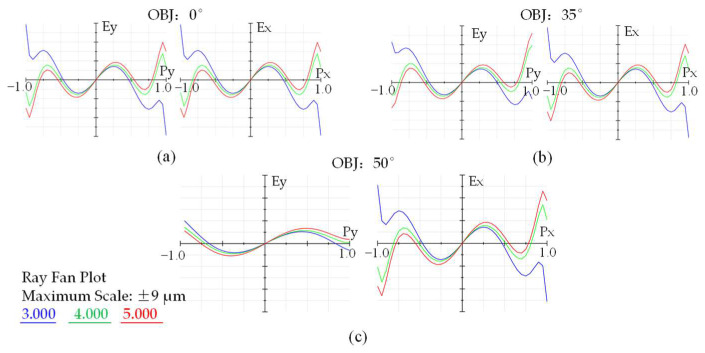
Ray fan plot of the field angle 0° (**a**), 35° (**b**), 50° (**c**), respectively.

**Figure 9:**
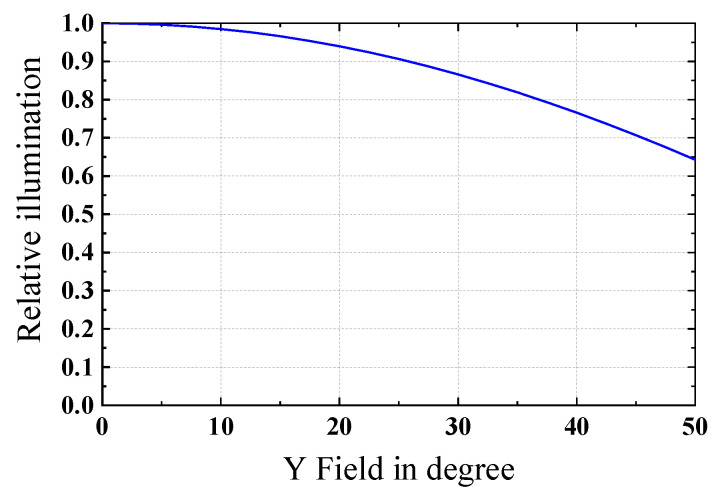
Relative illumination.

Although the imaging quality of the 0.7 FOV is allowed to decline to a certain extent, it can still meet the requirements of industrial production. The ray fan plots for the field angles of 0°, 35°, and 50° are shown in Figure 8a–c, respectively. The higher angle fans are partially vignetted but otherwise identical. The ray fan shows significant uncorrected spherical aberrations with a comparable amount of focus error, due to spherical and axial chromatic aberrations and spherochromatism. The effects of these aberrations are apparent in the MTF plot of Figure 7b. Figure 9 shows the relative illumination (RI) of the designed monocentric lens. The off-axis 35° FOV has a RI of 0.82, while the maximum off-axis FOV has a RI of 0.64. The Figure shows that the RI satisfies the use requirements.

## 4. Heatless Processing and Tolerance Analysis

### 4.1. Non-Thermalization Treatment

Infrared optical instruments are often used in extreme environments, due to their unique characteristics, which require systems that can maintain a good imaging quality over a wide temperature range. However, the glass materials that can be used for infrared optical systems are limited, and the refractive index temperature coefficient is larger and more susceptible to temperature changes compared to ordinary glass infrared materials [35]. Thus, infrared optical systems must be heatless. There are two commonly used non-thermalization technologies [36]: (1) mechanical active, which uses additional temperature measurement systems to achieve active focusing of the optical system through different temperature control motors; and (2) optical passive, which compares the optical coefficients of different optical materials, selects several suitable materials for combination, and counteracts the out-of-focus effects brought about by temperature through the different expansion coefficients of the materials.

The monocentric structure is so compact that mechanical activity is impossible, and the limited selection of materials makes optically passive heat dissipation an impractical method for realizing the system. To ensure the stability of the optical system, a primary thermally controlled thermostatic optoelectronic pod was used [37]. Considering the working temperature of the infrared material, a constant temperature pod with a range of 15–25 °C was selected for the cabin [38,39]. The optical system was optimized to achieve a better imaging effect compared to the original monocentric structure within the temperature control range of the constant temperature pod.

**Figure 10:**
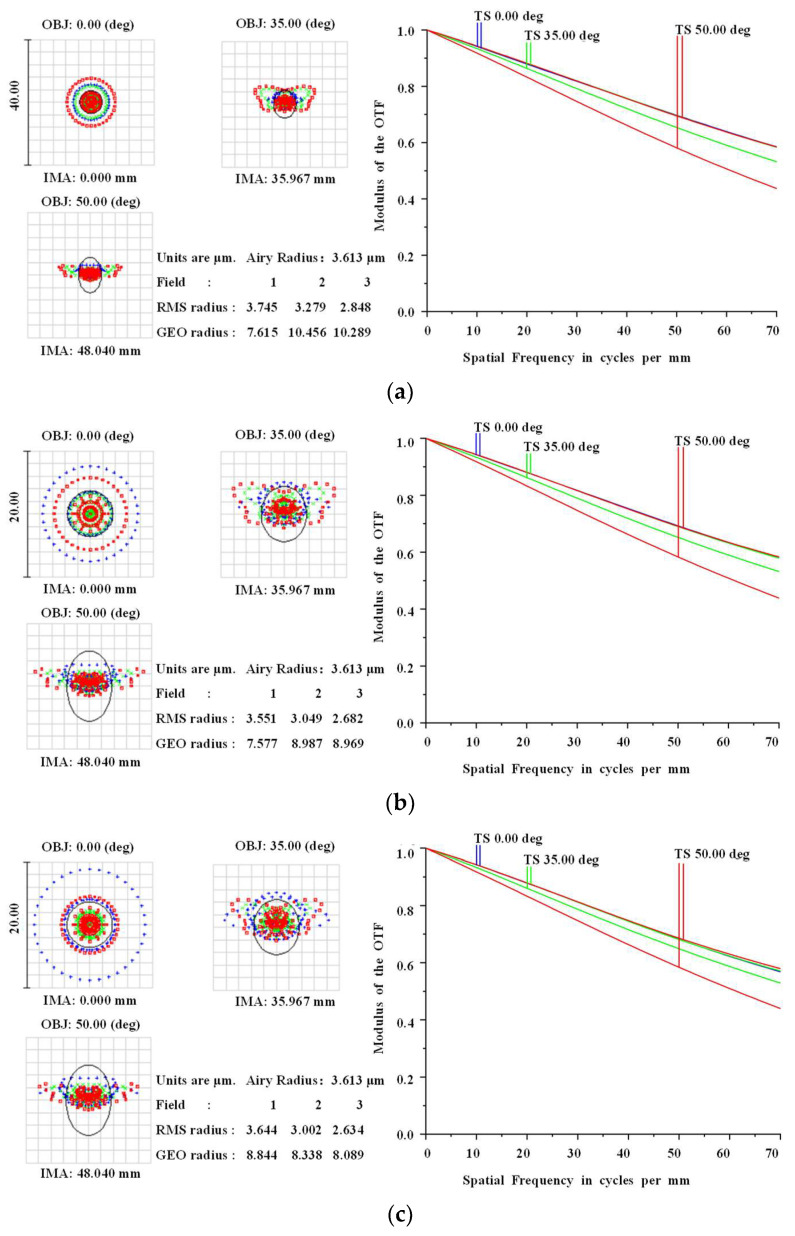
Spot and MTF diagrams of the system at 15 °C (**a**), 20 °C (**b**), and 25 °C (**c**).

Figure 10 shows the spot diagram and MTF curve diagrams after optimization. The simulation results for the system at 15 °C, 20 °C, and 25 °C are shown in Figure 10. As shown in the Figure, the MTF values of the system at the spatial frequency of 70 lp/mm were all greater than 0.4, and the spot diagram shows that the system has a good energy harvesting capability in different FOVs at different temperatures, and fulfilled the purpose of optimization.

### 4.2. Tolerance Analysis

A good optical design meets the requirements for technological processes and maximizes imaging quality. The design outcomes were examined using Zemax’s built-in tolerance analysis program, employing an MTF tolerance sensitivity analysis. The types of errors, including the glass refractive index deviation, component processing, and assembly tolerances, can degrade the performance of optical systems in production manufacturing. The sensitivity is set by the F-number of the objective and the radius of curvature of the objective image surface. The tolerance parameter values are listed in Table 3, to confirm the machinability of the monocentric lens. Monte Carlo simulation was used for 200 random error analyses; the results are shown in Table 4. After introducing the error, more than 90% of the samples at the Nyquist frequency of 70 lp/mm have MTF values greater than 0.316, which meets the requirements for industrial production.

## 5. Conclusions

According to the high demands of uncrewed aerial reconnaissance aircraft for airborne lenses, a wide FOV mid-wave infrared lens with a monocentric structure was designed, and the practicality of using monocentric lenses was realized by introducing sensor arrays in the absence of large-scale curved sensors. The structure has a response waveband of 3–5 μm, a FOV of 100°, a relative aperture of 1, and a total length of 124 mm, with a ground sampling resolution of 0.33 m. The requirements of a short total length, consistent imaging quality, and a wide-field were also met. We obtained an initial structure with five-glass (5GS) asymmetric monocentric lens with an air gap using ray-tracing and global optimization algorithms. This 5GS structure is more compact than the conventional symmetric structure and can simultaneously correct spherical and chromatic aberration, as well as spherochromatism. We also discussed the athermalisation method and tolerance analysis of the structure. The simulation results for the system error showed that the tolerance range of the design parameters was reasonable. The image quality meets the design specifications. By using primary thermal control, the temperature of the optoelectronic chamber was between 15 and 25 °C. The image plane with temperature drift still meets the requirements for spatial resolution in this temperature range without focusing. This paper can provide a reference for the design of airborne large aperture mid-wave infrared optical systems. With the development of science and technology, curved sensors with a large curvature will be fabricated, to make monocentric optical systems compact and wide-field, which will be able practically replace relay steering systems, sensor arrays, and fiber optic bundles, to reduce system complexity.

## Figures and Tables

**Table 1:** Optical design parameters.

Parameter	Value
Wave/μm	3–5
Focal length/mm	63.5
Total length/mm	100–150
FOV/(°)	100
F number	1

**Table 2:** Parameters of the monocentric lens.

Surface Type	Radius/mm	Thickness/mm	Glass Type
OBJ (0)	Infinity	Infinity	
1	61.445	43.764	GERMANIUM
2	17.681	17.681	AMTIR1
STO (3)	Infinity	23.159	AMTIR1
4	−20.158	18.253	ZNS_BROAD
5	−41.411	2.459	
6	−47.872	13.717	IG2
7	−53.657	5.102	
IMA (8)	−63.473		

**Table 3:** Tolerance analysis items and their values.

Item	Value
Refractive index Radius/mm Thickness/mm	0.0005 0.002 0.002
Element tilt/(′)	±5
Element decenter/mm	0.002
Irregularity	0.05 fringe
Abbe number	0.5%

**Table 4:** Results of the Monte Carlo simulation.

**Sampling Probability**	90%	80%	50%	20%	10%
**MTF Value**	0.316	0.337	0.399	0.453	0.483

## Data Availability

The data presented in this study are available on request from the corresponding author.
